# Supplementary material for: STUB1 is an intracellular checkpoint for interferon gamma sensing
Source: Sci Rep. 2022 Aug 18;12:14087. doi: 10.1038/s41598-022-18404-4 (PMC9388626; doi:10.1038/s41598-022-18404-4)

STUB1 is an intracellular checkpoint for interferon gamma sensing

Simon Ng[1], Shuhui Lim[1], Adrian Chong Nyi Sim[1], Ruban Mangadu[2], Ally Lau[3], Chunsheng Zhang[4], Sarah Bollinger Martinez[5], Arun Chandramohan[1], U-Ming Lim[3], Samantha Shu Wen Ho[6], Shih Chieh Chang[1], Pooja Gopal[1], Lewis Z. Hong[6], Adam Schwaid[5], Aaron Zefrin Fernandis[3], Andrey Loboda[4], Cai Li[7], Uyen Phan[2], Brian Henry[1, *], Anthony W. Partridge[1, *]

[1]Quantitative Biosciences, MSD, Singapore

[2]Discovery Oncology, Merck & Co., Inc., South San Francisco, CA, USA

[3]Target & Pathway Biology, MSD, Singapore

[4]Informatics, Merck & Co., Inc., Boston, MA, USA

[5]Chemical Biology, Merck & Co., Inc., Boston, MA, USA

[6]Translational Biomarkers, MSD, Singapore

[7]Quantitative Biosciences, Merck & Co., Inc., Boston, MA, USA

*Corresponding author: Brian Henry (brian.henry3@merck.com), Anthony W. Partridge (awpartridge@gmail.com)

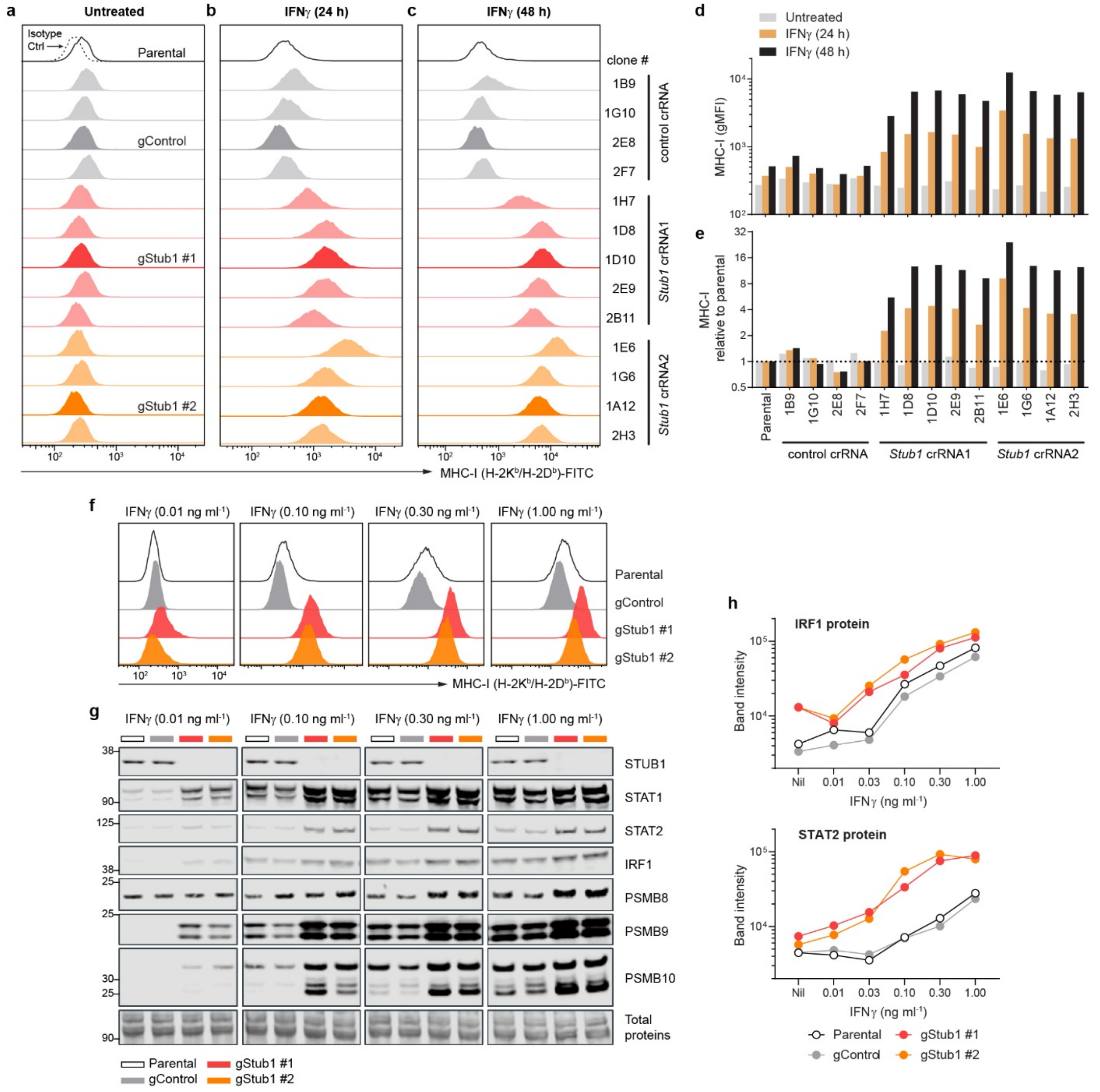

Supplementary Fig. 1 Loss of *Stub1* in B16-F10 melanoma increased the surface level of MHC-I and the protein level of STAT1, STAT2, IRF1, PSMB8, PSMB9 and PSMB10 in response to IFNγ. Related to Fig. 1. **a–e**, Flow cytometry analysis of cell surface level of MHC-I on parental B16-F10 and all CRISPR-edited clones isolated by single-cell subcloning (Supplementary Table 2). The tumour cells were either untreated (a) or treated with 0.10 ng ml^{-1} IFNγ for 24 h (b) or 48 h (c). The expression level of MHC-I on the tumour cells (d) and their relative abundance compared to the parental B16-F10 cells (e). All further experiments were performed using single-cell clone 2E8, 1D10 and 1A12 – termed gControl, gStub1 #1 and gStub1 #2 respectively. **f**, Flow cytometry analysis of cell surface MHC-I on parental, control or independent *Stub1*-null B16-F10 cells, following treatment with the indicated condition for 24 h. **g**, **h**, Western blot analysis of STUB1, STAT1, STAT2, IRF1, PSMB8, PSMB9 and PSMB10 in

tumour cells, following treatment with the indicated concentration of IFNγ for 24 h (g). Quantification of the protein level with LI-COR Image Studio (h). Band intensity was normalized with total protein signal. Representative of four (f) or two (g, h) independent experiments.

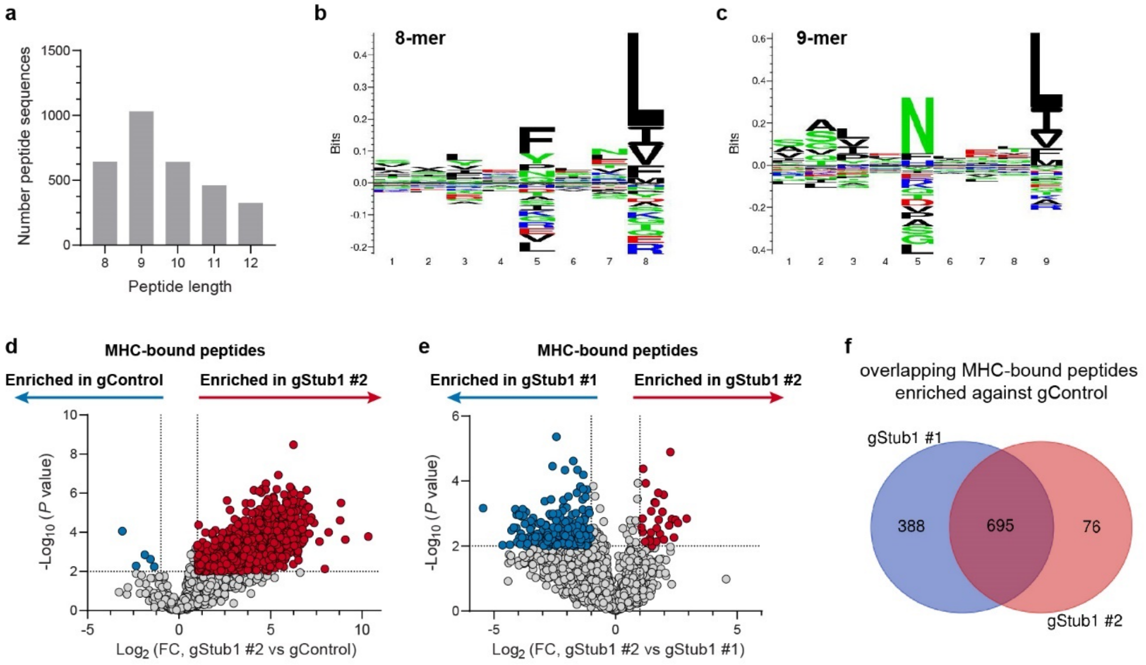

Supplementary Fig. 2 Immunopeptidomics of B16-F10 CRISPR cell lines. Related to Fig. 1g. **a**, Distribution of length for peptides identified by immunopeptidomics. **b**, **c**, Sequence motif of the 8- and 9-mers peptides identified by immunopeptidomics. **d**, **e**, Volcano plot showing differential presentation of MHC-associated peptide in the tumour cells, following stimulation with 0.10 ng ml^{-1} IFNγ for 24 hours. Red and blue circles highlight peptides significantly enriched in the respective tumour cells (2-fold cutoff, $P \leq 0.01$; $n = 3$ biological replicates). **f**, Venn diagram showing the number of unique peptides enriched for gStub1 #1 (1,083 peptides) and gStub1 #2 (771 peptides) relative to the gControl cells. There are 695 MHC-bound peptides overlapping between the two clonal *Stub1*-null cells.

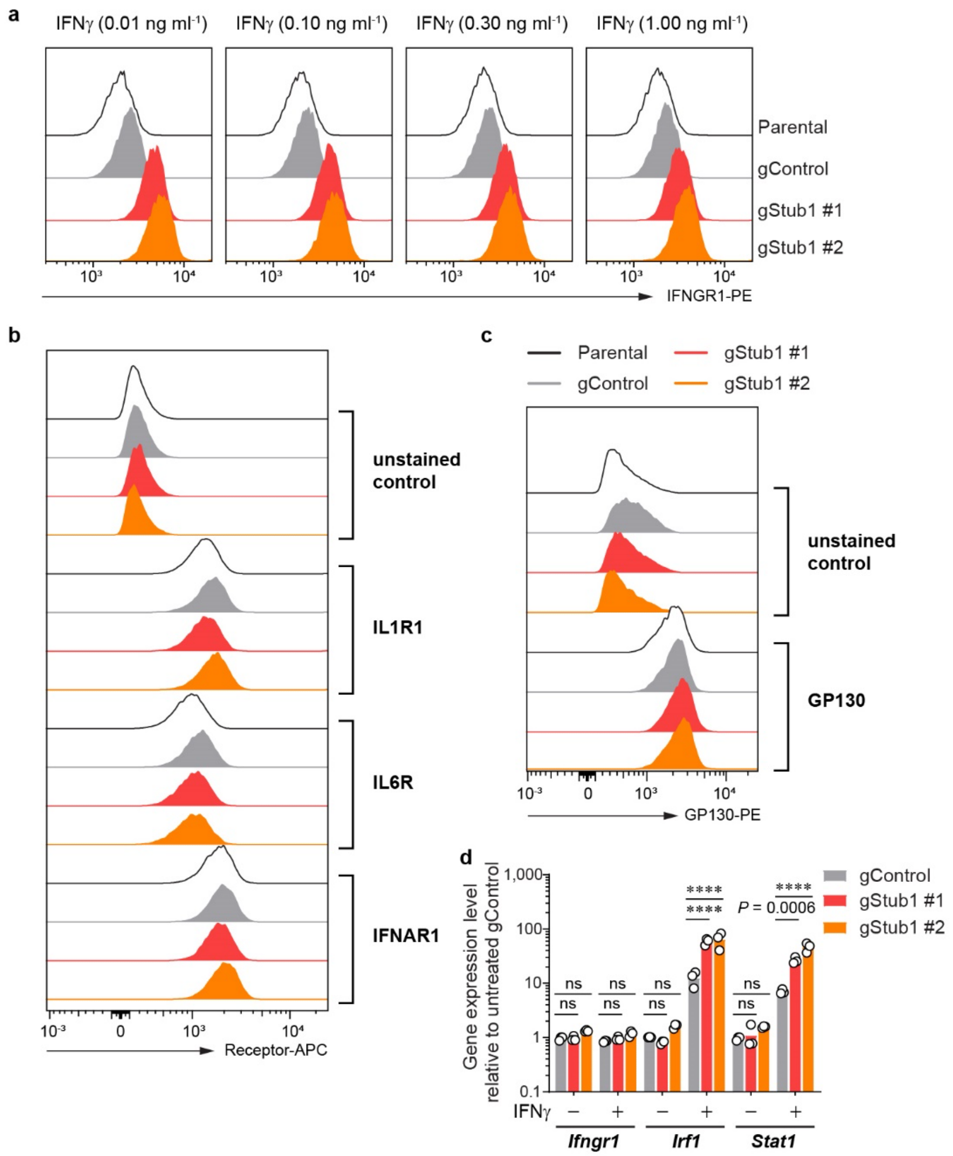

Supplementary Fig. 3 Analysis of the surface level of IFNGR1 and other immune-related receptors, and gene expression of *Ifngr1*, *Irf1* and *Stat1*. Related to Fig. 2. **a**, Flow cytometry analysis of cell surface IFNGR1 on parental, control or independent *Stub1*-null B16-F10 cells treated with IFNγ for 24 h. **b**, **c**, Flow cytometry analysis of cell surface IL1R1, IL6R or IFNAR1 (b) or GP130 (c) on parental, control or independent *Stub1*-null B16-F10 cells at resting state. **d**, qPCR analysis of the gene expression of *Ifngr1*, *Irf1* and *Stat1* relative to untreated gControl cells. Cells were stimulated with IFNγ (0.03 ng ml^{-1}) for 6 h. Expression level was normalized to a reference gene (*Tbp*). Data are mean with all data points from three independent experiments (d). *P* values were determined by ordinary two-way ANOVA in each transcribed gene with Dunnett's multiple comparisons test, **** $P \leq 0.0001$, ns $P > 0.98$ (d). Representative of three (a) or four (b–c) independent experiments.

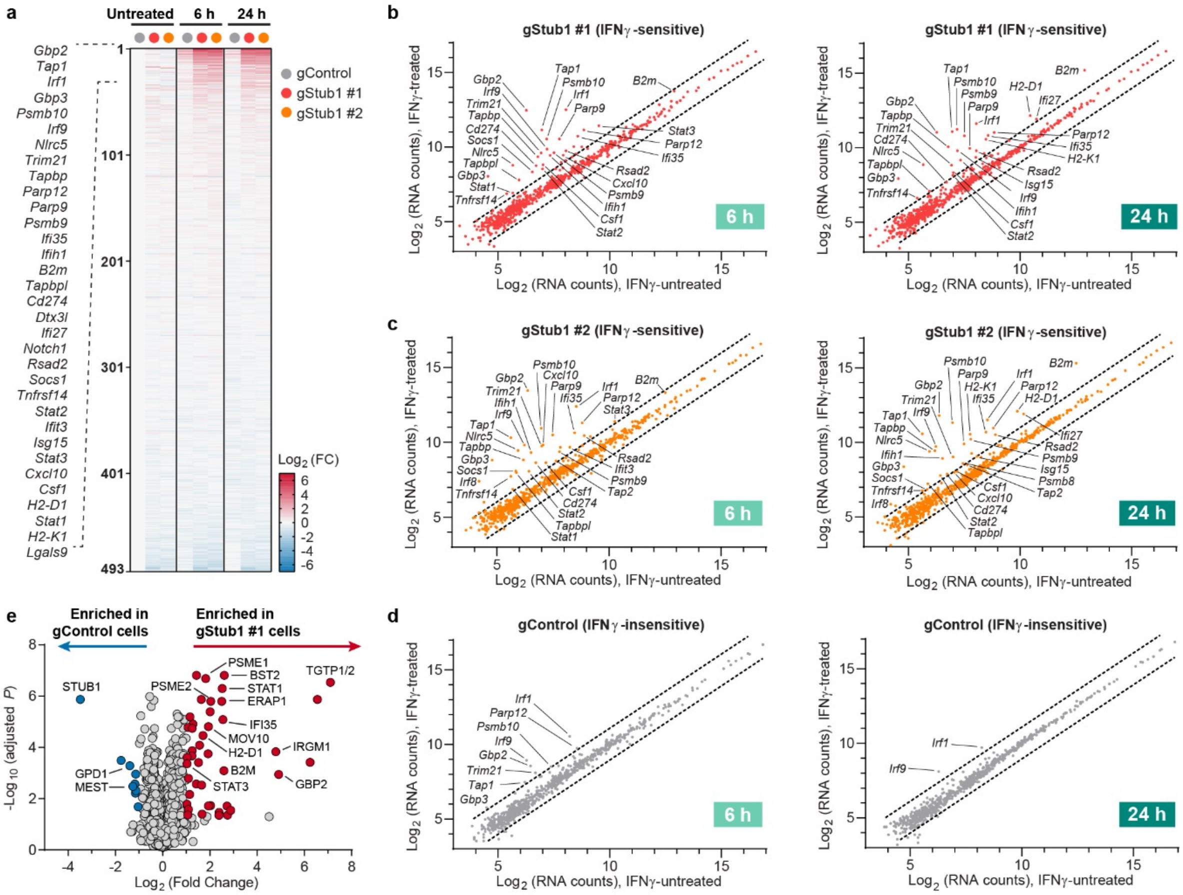

Supplementary Fig. 4 Loss of *Stub1* sensitized B16-F10 melanoma to IFNγ response as determined by gene expression profiling and proteome-wide studies. Related to Fig. 2. **a**, Heatmap showing relative gene expression profile of 493 out of 750 genes (NanoString PanCancer IO 360, Supplementary Table 3) in tumours cells which were either untreated or treated with 0.03 ng ml^{-1} IFNγ for 6 or 24 h. Weakly expressed genes (257 out of 750) were removed from the analysis. 33 genes upregulated by >2-fold in both *Stub1*-null cells relative to untreated gControl cells were listed explicitly (see Fig. 2e for the expanded heatmap). Refer to Method section for the description of fold change analysis. FC, fold change. **b–d**, Scatter plots showing the expression level of all 750 genes (Supplementary Table 3) in gControl cells (b) gStub1 #1 cells (c) or gStub1 #2 cells (d) before and after treatment with 0.03 ng ml^{-1} IFNγ for 6 h (left plot) or 24 h (right plot). Dotted lines depict the boundary of 2-fold change in the RNA counts which were normalized with 19 housekeeping genes. **e**, Volcano plot showing differential protein expression (Supplementary Table 4) in gStub1 #1 versus gControl cells, following treatment with 0.03 ng ml^{-1} IFNγ for 24 h. Red or blue circles highlight proteins being significantly enriched in gStub1 #1 or gControl cells respectively (2-fold cutoff, adjusted P ≤0.05; n = 6 replicates per cell group, 3 biological replicates × 2 MS replicates). Enriched proteins that overlap with gStub1 #2 cells (see Fig. 2f–g) are explicitly labeled in the plot. STUB1, GPD1 and MEST were consistently enriched in gControl cells as compared to either

gStub1 #1 or gStub1 #2 cells (see Fig 2f). Statistics details were described in proteomics method section.

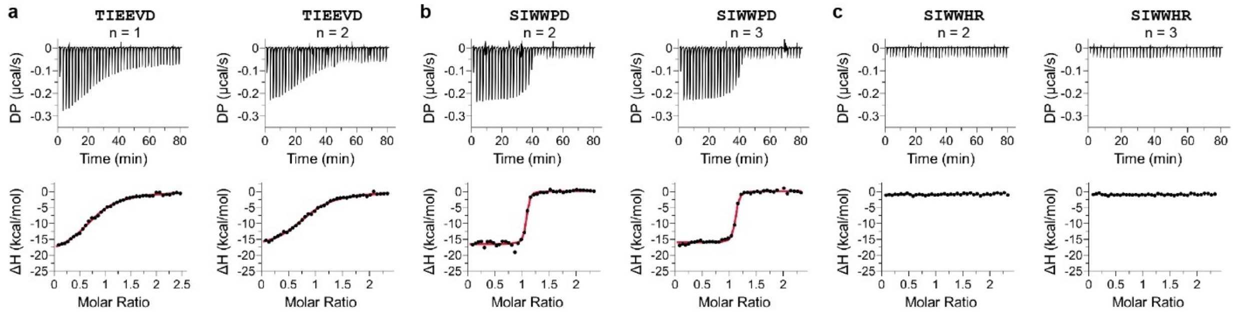

d

	TIEEVD	SIWWPD	SIWWHR
ΔT_m (°C)	5.2 ± 0.2	18.3 ± 0.1	-0.1 ± 0.2
IC_{50} (µM)	2.7 ± 0.2	0.34 ± 0.02	> 100
K_D (nM, ITC)	2190 ± 120	14 ± 2	>10,000
N (ITC)	0.81 ± 0.06	1.03 ± 0.07	NA
ΔH (kcal mol^{-1}, ITC)	-19.3 ± 1.7	-17.1 ± 1.2	NA
−TΔS (kcal mol^{-1}, ITC)	11.6 ± 1.8	6.3 ± 1.2	NA

Supplementary Fig. 5 Validation of the binding of synthetic peptides to STUB1 with multiple biophysical assays. Related to Fig. 3. **a–c**, Binding of the synthetic peptides to STUB1 (aa25–aa153) as determined by isothermal titration calorimetry (ITC). The peptides contain free carboxylic acid at the C-terminus and are acetylated at the N-terminus. Positive control peptide (TIEEVD) is derived from the C-terminal end of HSPA8 – the endogenous binding substrate of STUB1 (a). SIWWPD is bound strongly to the protein (b), whereas SIWWHR is a non-binding control (c). **d**, Summarized results from all biophysical assays. The shift in the melting temperature (ΔT_m) relative to the DMSO vehicle is reported as mean ± s.d. from three independent experiments. Half maximal inhibitory concentration (IC_{50}) is reported as mean ± s.e. derived from the 4-parameter sigmoidal curve fitted with the data of six replicates derived from two independent fluorescence polarization experiments. Dissociation constant (K_D), binding stoichiometry (N), enthalpy (ΔH) and entropy (-TΔS) are reported as mean ± s.d. from two (a) or three (b–c) independent ITC experiments.

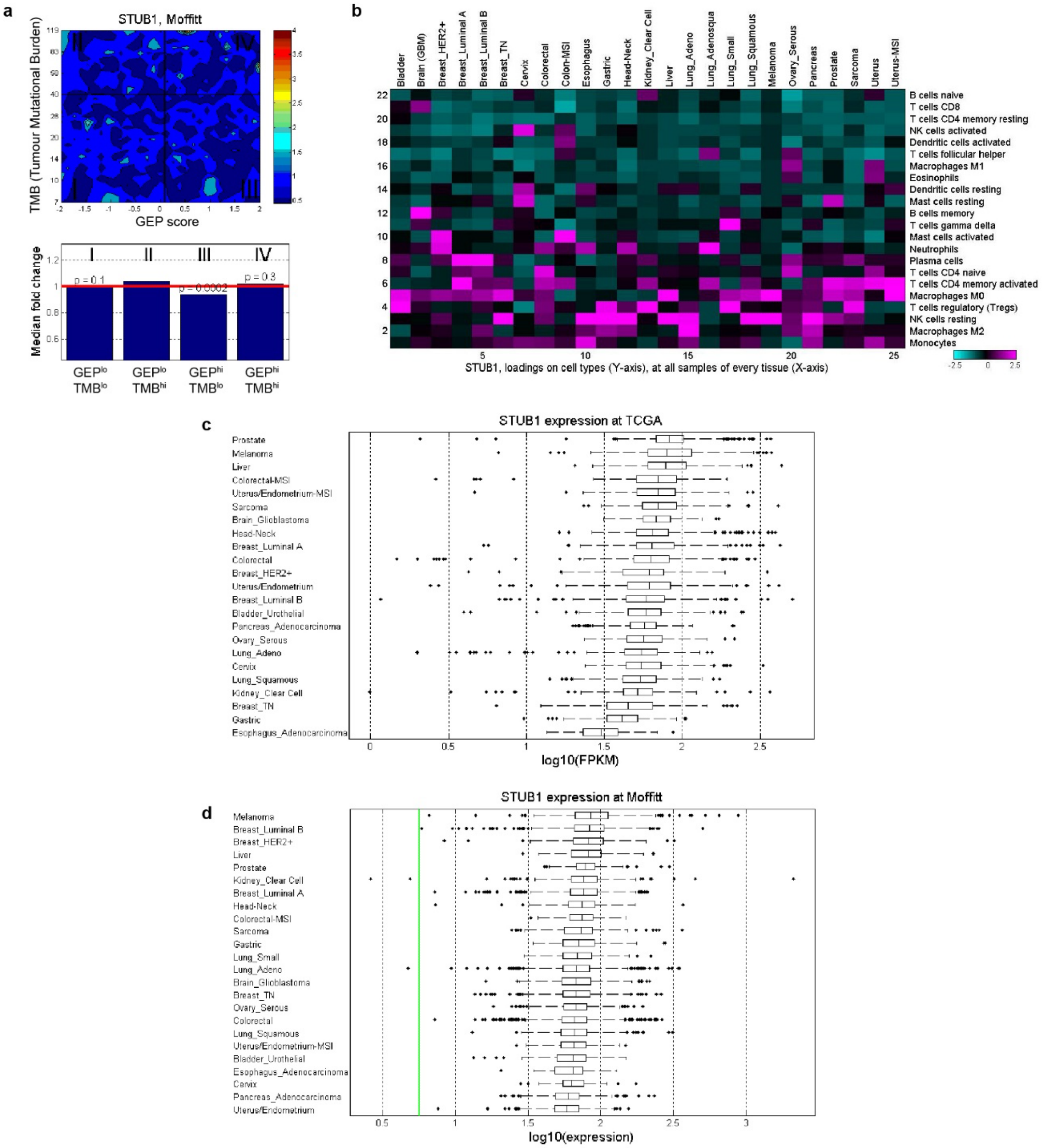

Supplementary Fig. 6 Correlation and expression of *STUB1* gene in Moffitt dataset. Related to Fig. 4. **a**, Contour plot illustrates the association of *STUB1* with TMB and GEP. Blue and red represent under- and overexpression, respectively. TMB cut-off was set at 40 and GEP cut-off corresponds to 55th percentile value for pan-cancer cohort. **b**, *In-silico* deconvolution analysis of bulk RNAseq data from Moffitt was used to establish the association between *STUB1* expression and different cell types. Deconvolution analysis, based on CIBERSORT, was performed separately for each tumor type. **c**, **d**, Relative *STUB1* expression level across major tumour tissues in TCGA (c) and Moffitt (d). Limit of detection >$\log_{10}(-1.7)$ in TCGA. The green line depicts the limit of detection (d).

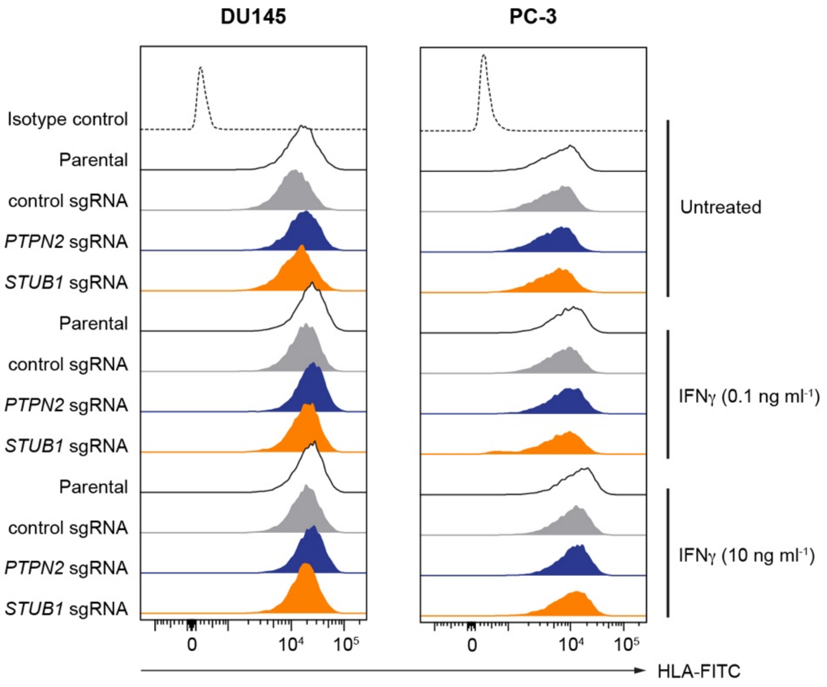

Supplementary Fig. 7 MHC-I expression of human tumour cells. Related to Fig. 5. Flow cytometry analysis of cell surface level of MHC-I (HLA-A,B,C) on parental DU145, parental PC-3 and the sgRNA-edited cells. The tumour cells were either untreated or treated with 0.10 or 10 ng ml^{-1} IFNγ for 24 h before being harvested for analysis.

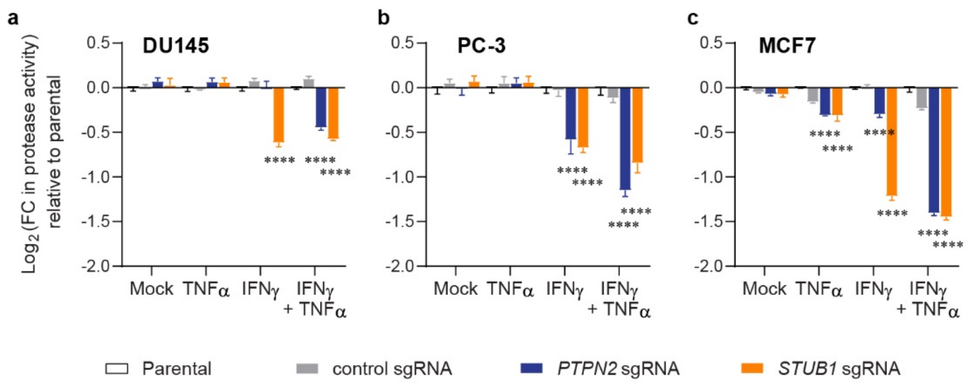

Supplementary Fig. 8 Inactivation of *STUB1* or *PTPN2* sensitized human tumour cells to growth inhibition induced by cytokines. Related to Fig. 5d. **a–c**, Fold change (FC) in live-cell protease activity relative to the parental cells as a quantification of viable cells. Measurements were performed using CellTiter-Fluor assay after 6-day treatment of DU145 (a), PC-3 (b), or MCF7 (c) cells and their corresponding CRISPR-edited lines with the cytokines (10 ng ml^{-1} each). Data are mean ± s.e.m. from three biological replicates (a–c). *P* values were determined by ordinary two-way ANOVA on Log2-transformed data with Dunnett's multiple comparisons test versus parental cells, ** *P* ≤0.01, **** *P* ≤0.0001, ns *P* >0.90 (a–c).

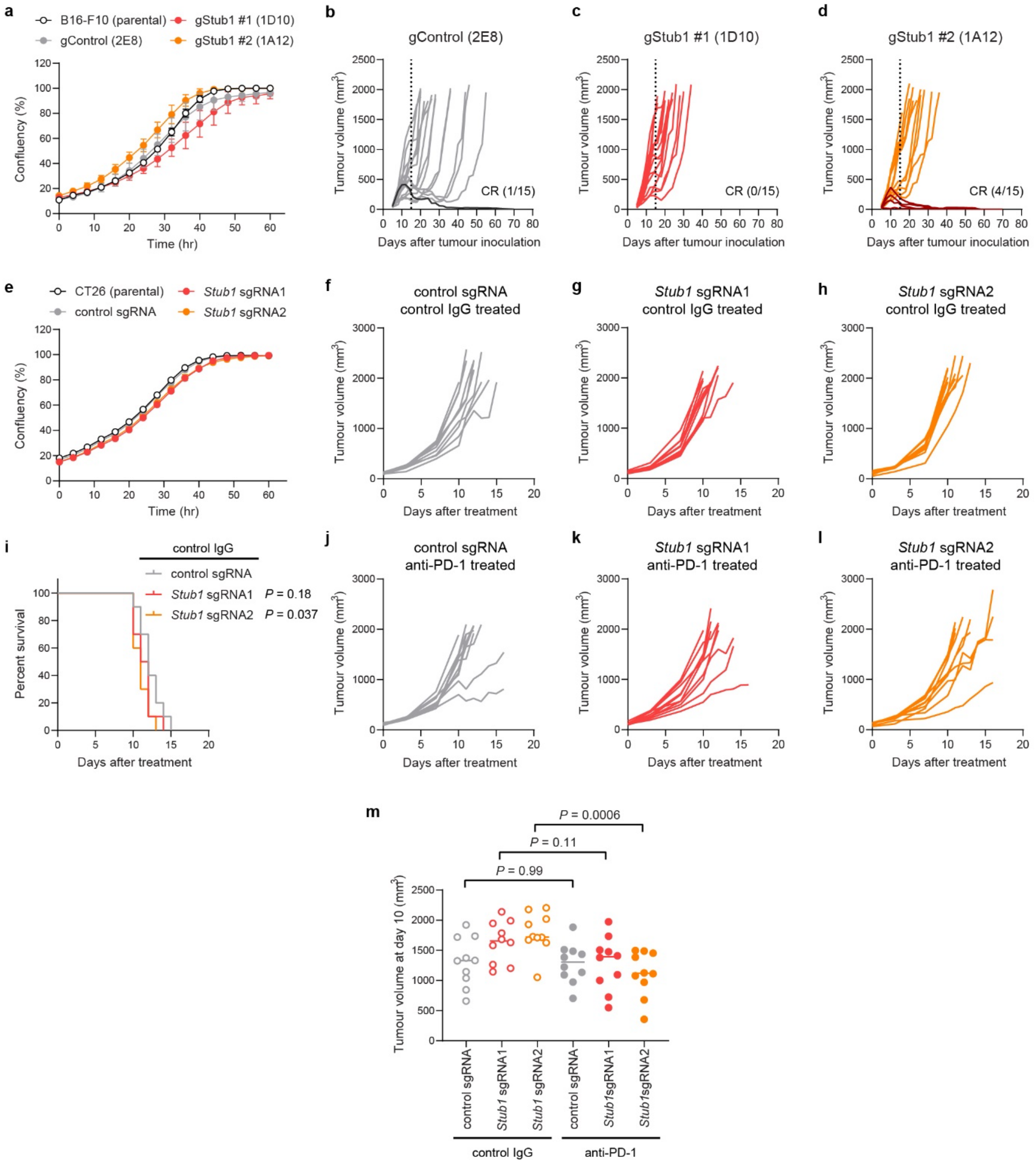

Supplementary Fig. 9 Additional data for the studies of syngeneic mouse models. Related to Fig. 6. **a**, Plot showing *in vitro* cellular growth kinetic of B16-F10 and the CRISPR clonal lines in standard 2D culture as measured by Incucyte (n = 3 biological replicates per cell type). **b–d**, Plot showing individual tumour volume of the CRISPR-edited B16-F10 clonal cells implanted into syngeneic mice (n = 15). CR, complete response. Dotted line indicates the day when mice received the last dose of anti-PD-1 antibody. **e**, Plot showing *in vitro* cellular growth kinetic of CT26 and the CRISPR lines in standard 2D culture as measured by Incucyte (n = 3 biological replicates per cell type). **f–h**, Plot showing individual tumour volume of the CRISPR-edited

CT26 cells implanted into syngeneic mice (*n* = 10) treated with control antibody. **i**, Kaplan-Meier survival curves of tumour-bearing mice treated with control antibody. Median survival: control sgRNA, 12 days; *Stub1* sgRNA1, 11.5 days, *Stub1* sgRNA2, 11 days. **j–l**, Plot showing individual tumour volume of the CRISPR-edited CT26 cells implanted into syngeneic mice (*n* = 10) treated with anti-PD-1 antibody. **m**, Plot showing tumour volume at day 10 for the CRISPR-edited CT26 cells implanted into mice (*n* = 10) treated with either control or anti-PD-1 antibody. Representative of two independent experiments (a, e). *P* values were determined by Log-rank (Mantel-Cox) test versus control tumours (i). *P* values were determined by two-way ANOVA with Sidak's multiple comparisons test (m). Data are mean with all data points derived from 10 mice per group (m).

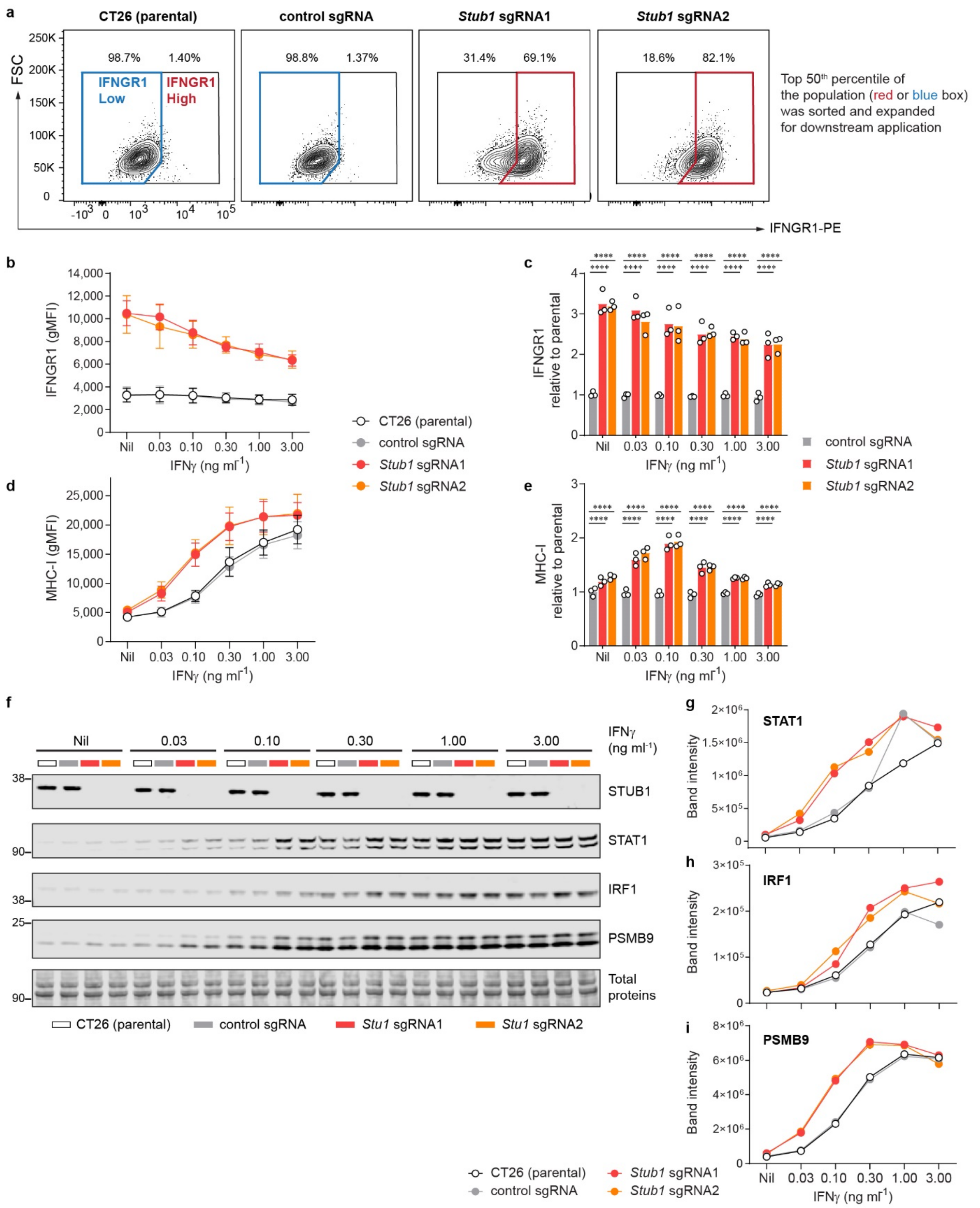

Supplementary Fig. 10 Loss of *Stub1* in CT26 tumour cells elevated the surface level of IFNGR1, leading to increased expression of MHC-I, STAT1, IRF1 and PSMB9 in response to IFNγ. Related to Fig. 6d–f and Supplementary Fig. 8e–m. **a**, Flow cytometry 2D plot showing the expression level of IFNGR1 on the cellular surface of CT26 and the corresponding CRISPR-

edited cells. We gated for the population with high expression of IFNGR1 and sorted for the top 50th percentile in the gated population (red box) to enrich the *Stub1*-null cells. Similar gating (IFNGR1 Low) and sorting (top 50th percentile, blue box) strategies were consistently applied to the parental and control sgRNA targeting CT26 cells. **b–e**, Flow cytometry analysis of cell surface IFNGR1 (b, c) and MHC-I (d, e) expressed on parental, control or independent *Stub1*-null CT26 cells (sorted). gMFI, geometric mean fluorescence intensity. **f–i**, Western blot analysis of the expression level of STUB1, STAT1, IRF1, and PSMB9 in parental, control or independent *Stub1*-null CT26 cells (sorted). Band intensities were quantified with LI-COR Image Studio and normalized with total protein signal. The tumour cells were either untreated (Nil) or treated with recombinant mouse IFNγ for 24 h (b–i). Data are mean ± s.d. (b, d) or mean with all data points (c, e) from three independent experiments. *P* values were determined by ordinary two-way ANOVA on Log2-transformed data with Dunnett's multiple comparisons test, **** $P \leq 0.0001$ (c, e). Representative of two independent experiments (f–i).

Supplementary Table S1–4 are available as separate files (.xlsx) in supplementary information.

Supplementary Table S1. Summary of genetic screens that have uncovered STUB1 as a regulator of immune pathway.

Supplementary Table S2. Details of single clone isolated from *Stub1*-knockout B16-F10 cells.

Supplementary Table S3. Normalized count and fold change of NanoString gene expression data.

Supplementary Table S4. Protein abundance and differential enrichment of proteomics data.

Supplementary Table S5. DNA coding and protein sequences of the inhibitory biologics and its control.

Construct name	Abbrevia-tion	DNA coding sequences	Protein sequences
FLAG-mCherry2-GGSGGS-SIWWPD	mCherry2-SIWWPD	ATGGATTACAAGGACGACGACGACAAGGCTA<br>GCGTGAGCAAGGGCGAGGAGGATAACATGGC<br>CATCATCAAGGAGTTCATGCGCTTCAAGGTGC<br>ACATGGAGGGCTCCGTGAACGGCCACGAGTT<br>CGAGATCGAGGGCGAGGGCGAGGGCCGCCCC<br>TACGAGGGCACCCAGACCGCCAAGCTGAAGG<br>TGACCAAGGGTGGCCCCCTGCCCTTCGCCTGG<br>GACATCCTGTCCCCTCAGTTCATGTACGGCTC<br>CAAGGCCTACGTGAAGCACCCCGCCGACATC<br>CCCGACTACTTGAAGCTGTCCTTCCCCGAGGG<br>CTTCAATTGGGAGCGCGTGATGAACTTCGAG<br>GACGGCGGCGTGGTGACCGTGACCCAGGACT<br>CCTCCCTGCAGGACGGCGAGTTCATCTACAAG<br>GTGAAGCTGCGCGGCACCAACTTCCCCTCCGA<br>CGGCCCCGTAATGCAGTGTCGTACCATGGGCT<br>GGGAGGCCTCCACTGAGCGGATGTACCCCGA<br>GGACGGCGCCCTGAAGGGCGAGATCAAGCAG<br>AGGCTGAAGCTGAAGGACGGCGGCCACTACG<br>ACGCTGAGGTCAAGACCACCTACAAGGCCAA<br>GAAGCCCGTGCAGCTGCCCGGCGCCTACAAC<br>GTCGACATCAAGTTGGACATCCTTTCCCACAA<br>CGAGGACTACACCATCGTGGAACAGTACGAA<br>CGCGCCGAGGGCCGCCACTCCACCGGCGGCA<br>TGGACGAGCTGTACAAGGGAGGTAGCGGGGG<br>TAGTTCCATATGGTGGCCTGACTGA	MDYKDDDDKASVSKG<br>EEDNMAIIKEFMRFKV<br>HMEGSVNGHEFEIEGE<br>GEGRPYEGTQTAKLKV<br>TKGGPLPFAWDILSPQF<br>MYGSKAYVKHPADIPD<br>YLKLSFPEGFNWERVM<br>NFEDGGVVTVTQDSSL<br>QDGEFIYKVKLRGTNFP<br>SDGPVMQCRTMGWEA<br>STERMYPEDGALKGEI<br>KQRLKLKDGGHYDAE<br>VKTTYKAKKPVQLPGA<br>YNVDIKLDILSHNEDYT<br>IVEQYERAEGRHSTGG<br>MDELYKGGSGGSSIW<br>WPD
FLAG-mCherry2-GGSGGS-SIWWHR	mCherry2-SIWWHR	ATGGATTACAAGGACGACGACGACAAGGCTA<br>GCGTGAGCAAGGGCGAGGAGGATAACATGGC<br>CATCATCAAGGAGTTCATGCGCTTCAAGGTGC<br>ACATGGAGGGCTCCGTGAACGGCCACGAGTT<br>CGAGATCGAGGGCGAGGGCGAGGGCCGCCCC<br>TACGAGGGCACCCAGACCGCCAAGCTGAAGG<br>TGACCAAGGGTGGCCCCCTGCCCTTCGCCTGG<br>GACATCCTGTCCCCTCAGTTCATGTACGGCTC<br>CAAGGCCTACGTGAAGCACCCCGCCGACATC<br>CCCGACTACTTGAAGCTGTCCTTCCCCGAGGG<br>CTTCAATTGGGAGCGCGTGATGAACTTCGAG<br>GACGGCGGCGTGGTGACCGTGACCCAGGACT<br>CCTCCCTGCAGGACGGCGAGTTCATCTACAAG<br>GTGAAGCTGCGCGGCACCAACTTCCCCTCCGA<br>CGGCCCCGTAATGCAGTGTCGTACCATGGGCT<br>GGGAGGCCTCCACTGAGCGGATGTACCCCGA<br>GGACGGCGCCCTGAAGGGCGAGATCAAGCAG<br>AGGCTGAAGCTGAAGGACGGCGGCCACTACG<br>ACGCTGAGGTCAAGACCACCTACAAGGCCAA<br>GAAGCCCGTGCAGCTGCCCGGCGCCTACAAC<br>GTCGACATCAAGTTGGACATCCTTTCCCACAA<br>CGAGGACTACACCATCGTGGAACAGTACGAA<br>CGCGCCGAGGGCCGCCACTCCACCGGCGGCA<br>TGGACGAGCTGTACAAGGGAGGTAGCGGGGG<br>TAGTTCCATATGGTGGCATCGATGA	MDYKDDDDKASVSKG<br>EEDNMAIIKEFMRFKV<br>HMEGSVNGHEFEIEGE<br>GEGRPYEGTQTAKLKV<br>TKGGPLPFAWDILSPQF<br>MYGSKAYVKHPADIPD<br>YLKLSFPEGFNWERVM<br>NFEDGGVVTVTQDSSL<br>QDGEFIYKVKLRGTNFP<br>SDGPVMQCRTMGWEA<br>STERMYPEDGALKGEI<br>KQRLKLKDGGHYDAE<br>VKTTYKAKKPVQLPGA<br>YNVDIKLDILSHNEDYT<br>IVEQYERAEGRHSTGG<br>MDELYKGGSGGSSIW<br>WHR

Uncropped Western Blot image

Fig. 1d

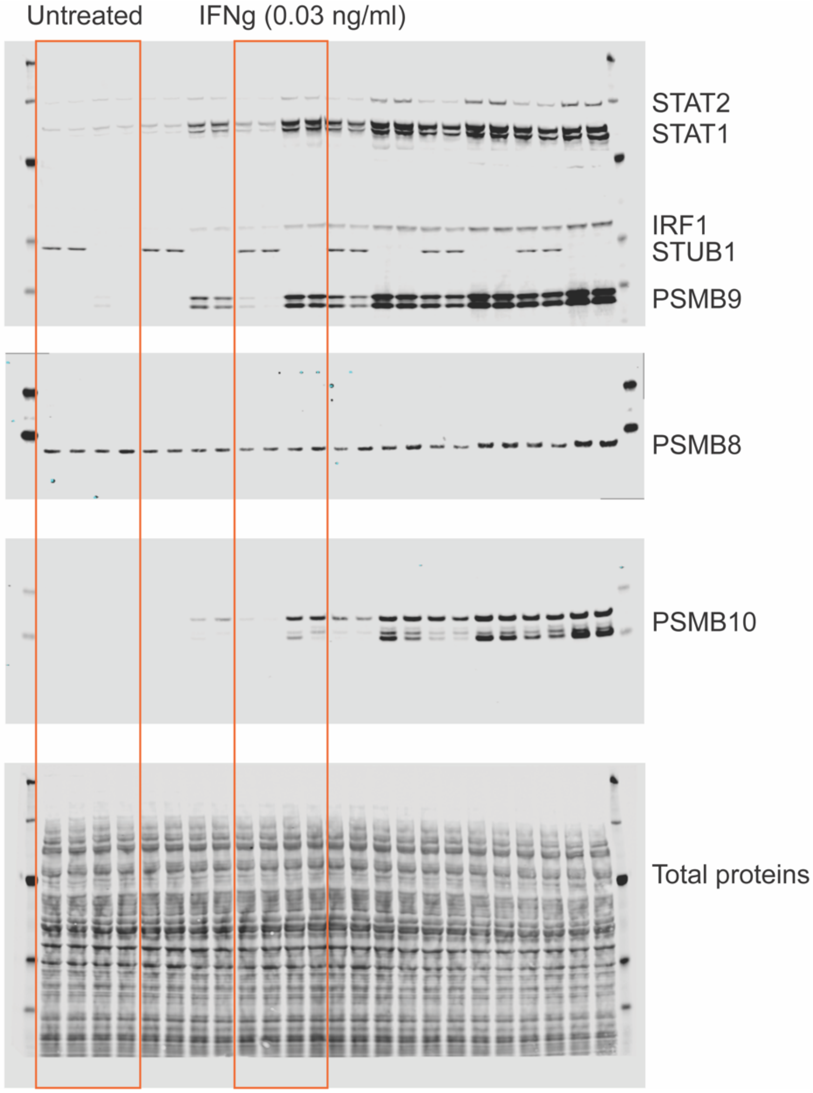

Fig. 1f

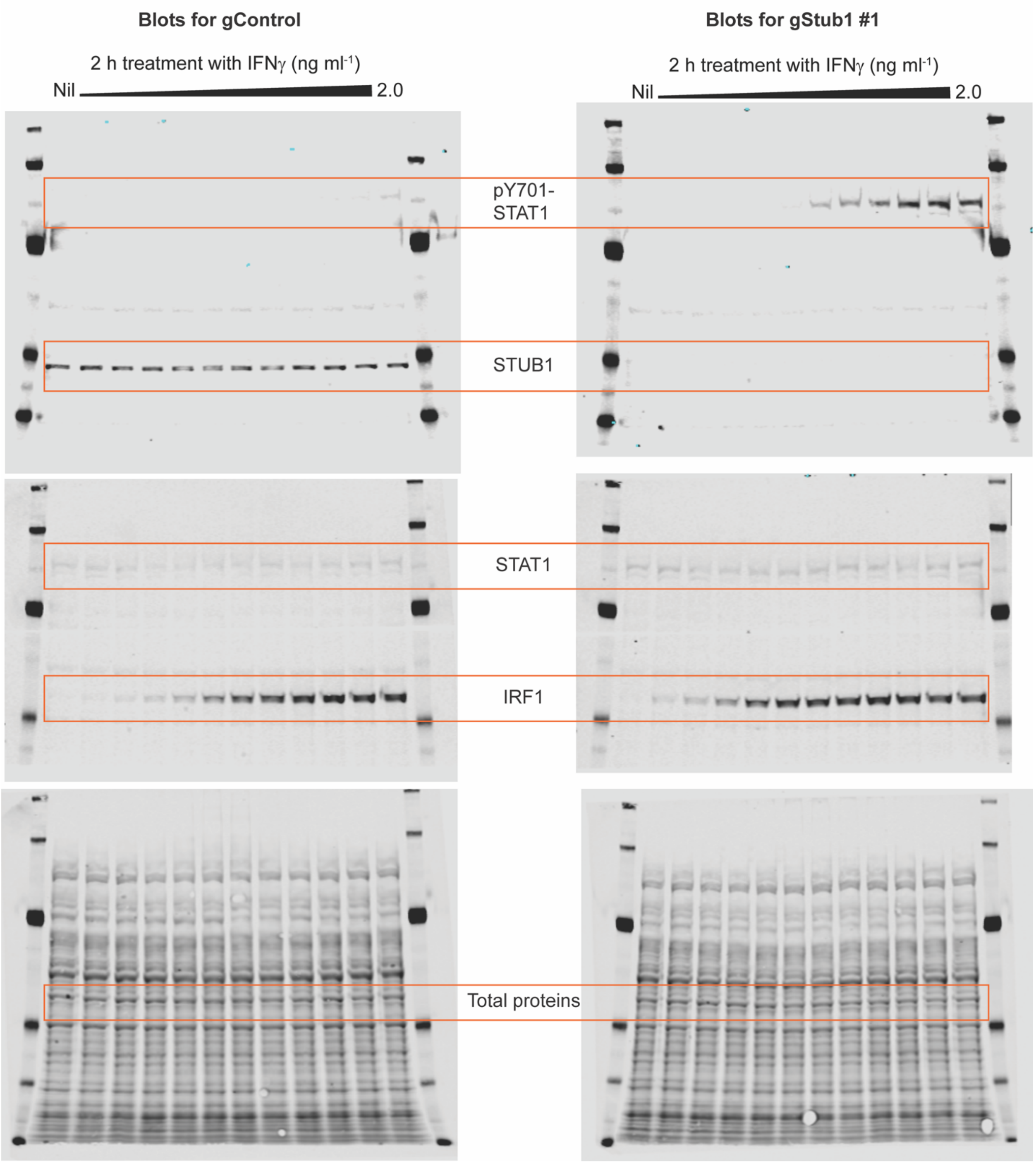

Blots for gControl
2 h treatment with IFNγ (ng ml-1)
Nil
2.0
Blots for gStub1 #1
2 h treatment with IFNγ (ng ml-1)
Nil
2.0
pY701-
STAT1
STUB1
STAT1
IRF1
Total proteins

Fig. 3h

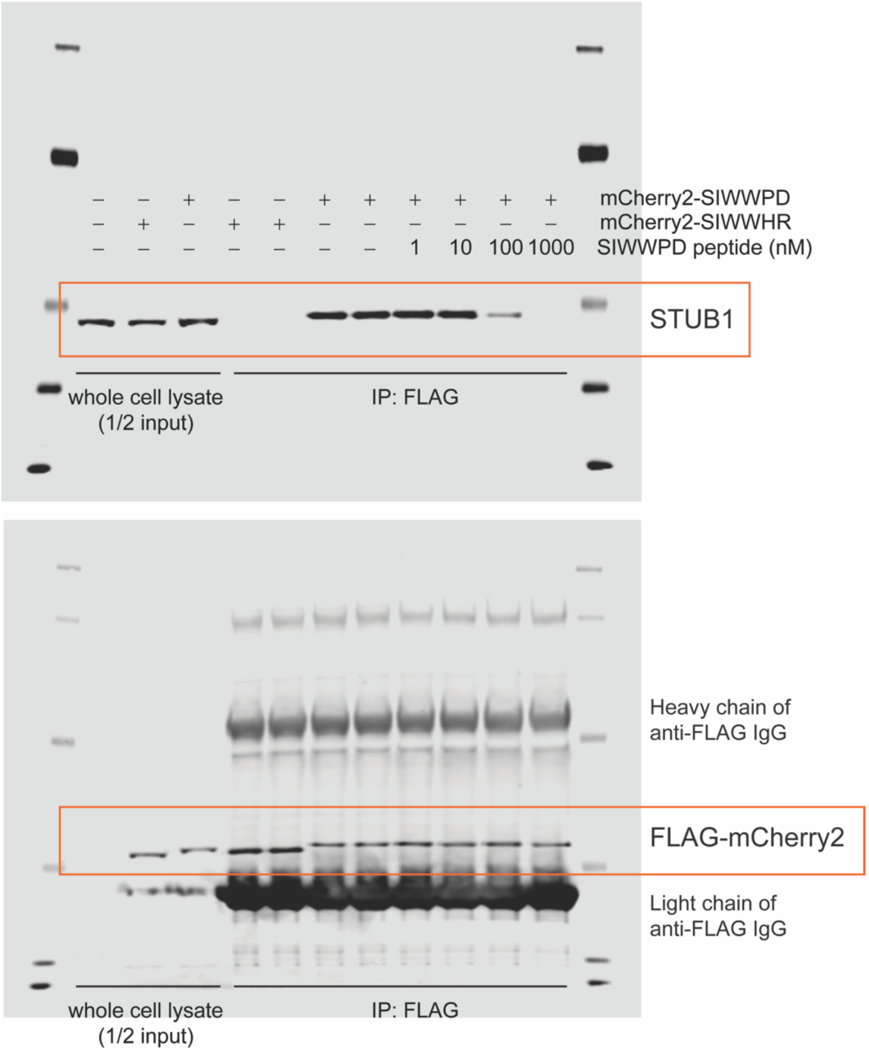
– – + – – + + + + + + mCherry2-SIWWPD
– + – + + – – – – – – mCherry2-SIWWHR
– – – – – – – 1 10 100 1000 SIWWPD peptide (nM)
STUB1
whole cell lysate (1/2 input)
IP: FLAG
Heavy chain of anti-FLAG IgG
FLAG-mCherry2
Light chain of anti-FLAG IgG
whole cell lysate (1/2 input)
IP: FLAG

Fig. 5a

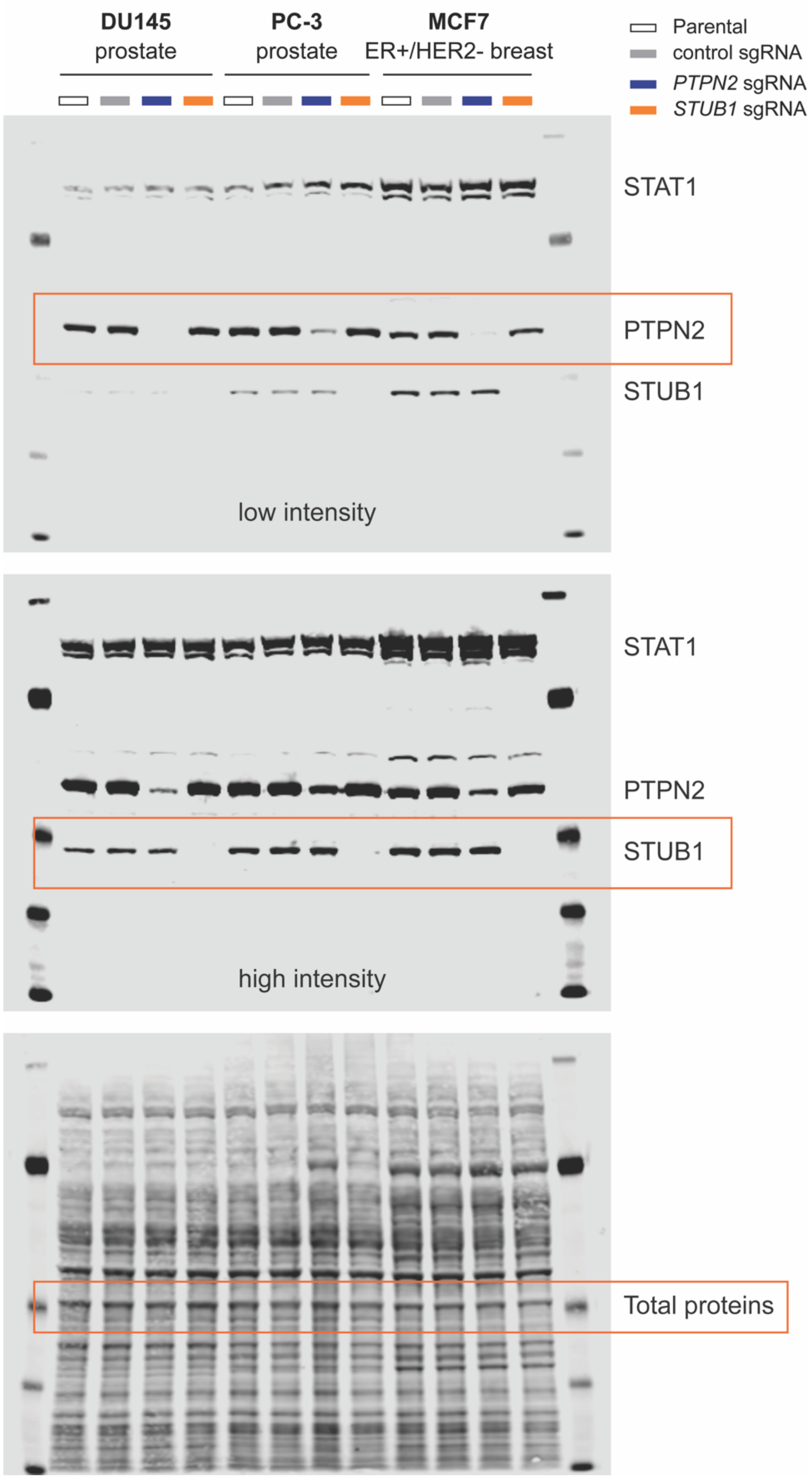
DU145
prostate
PC-3
prostate
MCF7
ER+/HER2- breast
Parental
control sgRNA
PTPN2 sgRNA
STUB1 sgRNA
STAT1
PTPN2
STUB1
low intensity
STAT1
PTPN2
STUB1
high intensity
Total proteins

Supplementary Fig. 1g

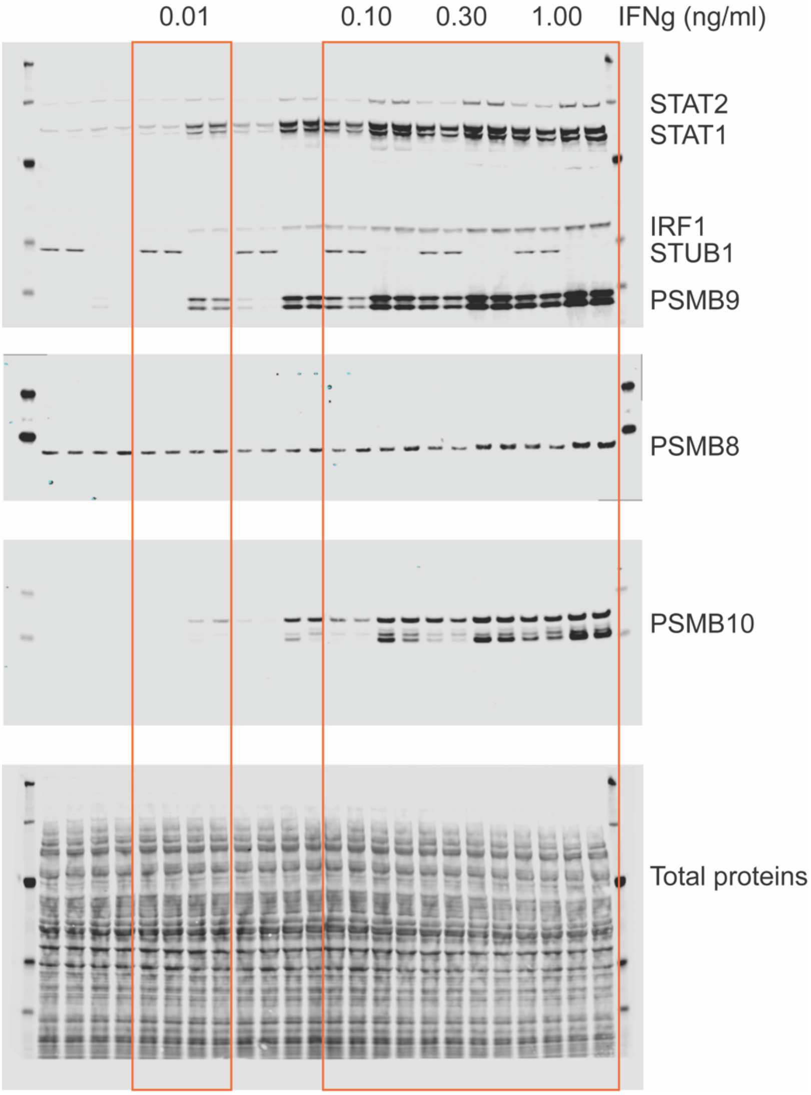

Supplementary Fig. 10f

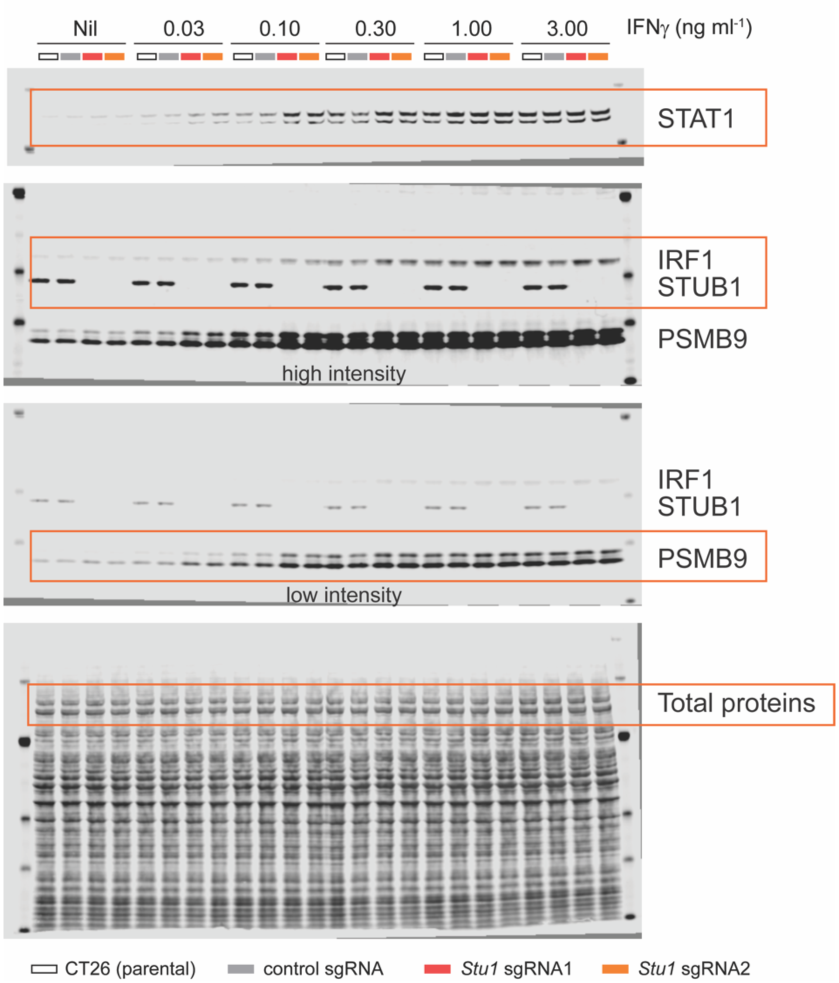

Supplement: Supplementary file 6 — Supplementary Information 6. [file 41598_2022_18404_MOESM6_ESM.pdf]
